# Circadian rhythm of intraocular pressure

**DOI:** 10.1186/s12576-024-00905-8

**Published:** 2024-03-02

**Authors:** Keisuke Ikegami

**Affiliations:** Laboratory of Regulation in Metabolism and Behavior, Faculty of Agriculture, Kyushu University, 744 Motooka, Nishi-Ku, Fukuoka, 819-0395 Japan

**Keywords:** Circadian rhythm, Glucocorticoids, Sympathetic nerve system, Aqueous humor, Ciliary body, Trabecular meshwork

## Abstract

Intraocular pressure (IOP) plays a crucial role in glaucoma development, involving the dynamics of aqueous humor (AH). AH flows in from the ciliary body and exits through the trabecular meshwork (TM). IOP follows a circadian rhythm synchronized with the suprachiasmatic nucleus (SCN), the circadian pacemaker. The SCN resets peripheral clocks through sympathetic nerves or adrenal glucocorticoids (GCs). IOP's circadian rhythm is governed by circadian time signals, sympathetic noradrenaline (NE), and GCs, rather than the local clock. The activity of Na^+^/K^+^-ATPase in non-pigmented epithelial cells in the ciliary body can influence the nocturnal increase in IOP by enhancing AH inflow. Conversely, NE, not GCs, can regulate the IOP rhythm by suppressing TM macrophage phagocytosis and AH outflow. The activation of the β1-adrenergic receptor (AR)-mediated EPAC-SHIP1 signal through the ablation of phosphatidylinositol triphosphate may govern phagocytic cup formation. These findings could offer insights for better glaucoma management, such as chronotherapy.

## Background

Circadian clocks are highly conserved in most of living organisms, regulating approximately 24-h (circadian) rhythms in various physiological and behavioral processes, including sleep–wake cycles, endocrine systems, and metabolism. The suprachiasmatic nucleus (SCN), a paired structure in the anterior hypothalamus located above the optic chiasma, acts as a circadian pacemaker in mammals. Circadian rhythms are generated by a transcription–translation feedback loop that controls the expression of clock genes [1]. The Circadian Locomotor Output Cycles Kaput (Clock) protein and Brain and Muscle Arnt-Like Protein 1 (Bmal1) heterodimerize to form a transcriptional activator complex, which activates the Period (Per) and Cryptochrome (Cry) repressor genes. The SCN receives light information from the retina through the retinohypothalamic tract and governs daily rhythms throughout the body [2]. Most peripheral tissues and cells synchronize with the SCN through various pathways, primarily involving the autonomic nervous system and endocrine signals [3]. However, the entrainment mechanism has not been fully elucidated due to its complexity.

Glaucoma is a leading cause of blindness in the elderly, but there is currently no effective cure. Abnormal intraocular pressure (IOP) inside the eye, such as high IOP, contributes to the development and progression of glaucoma, resulting in vision loss [4]. IOP is balanced by the aqueous humor (AH) and follows a circadian rhythm.

In AH dynamics, the non-pigmented epithelial cell (NPE) of the ciliary body plays a role in AH production [5]. Na^+^/K^+^-ATPase (NKA) and carbonic anhydrase (CA) are ion transporters that are crucial for maintaining osmotic pressure during AH inflow. CA, a zinc metalloenzyme, converts CO_2_ to HCO_3_^−^, generating osmotic gradient. Inhibiting NKA in the ciliary process using cardiac glycosides, such as ouabain, reduces the rate of AH production and IOP in both humans and experimental animals. Active transport of sodium is the primary driving force for AH secretion.

In contrast, the trabecular outflow pathway is responsible for homeostatic regulation of IOP and is controlled by the coordinated generation of AH outflow resistance mediated by the constituent cells of the trabecular meshwork (TM) and Schlemm’s canal (SC) [6, 7]. Additionally, uveoscleral outflow is known to be involved in human IOP regulation [8]. Phagocytosis of TM macrophage can reduce particulate material and debris in the AH, lowering outflow resistance, and contributing to decreased IOP [6]. Furthermore, actin remodeling, such as fragmentation or polymerization, regulates AH outflow, and the actin cytoskeleton of TM cells is a therapeutic target in patients with glaucoma. Small GTPase Rho-associated coiled-coil-containing protein kinase (ROCK) inhibitors lower IOP not only by relaxing the TM through the disruption of actin stress fibers but also by activating phagocytosis [9–11].

## Main text

### Glaucoma and IOP rhythm

In humans, nocturnal IOP increases regardless of posture [12]. IOP is also elevated at night in both nocturnal or diurnal animals [13] and in mice, it is regulated by the SCN [14]. Nocturnal IOP is also elevated in patients with glaucoma [6, 9], and IOP rhythms undergo phase shifts in patients with primary open-angle glaucoma (POAG) and normal-tension glaucoma [15]. It has been reported that normal-tension glaucoma is associated with an abnormal IOP rhythm [16]. Aging desynchronizes the IOP rhythm in healthy older subjects [17]. Interestingly, the IOP rhythm was disrupted in night-shift workers [18]. A recent study has reported that disrupted circadian IOP rhythms can lead to optic nerve damage and increase the risk of glaucoma [19]. Therefore, the regulation of the IOP rhythm, especially during the night, is crucial for glaucoma management, and the circadian mechanism in AH dynamics plays a significant role in glaucoma therapy. In a previous study, we demonstrated a dual pathway through which both norepinephrine (NE) and glucocorticoids (GCs) transmit timing information to the eye, forming the IOP rhythm in mice [20].

### Circadian time signal pathways

**Fig. 1:**
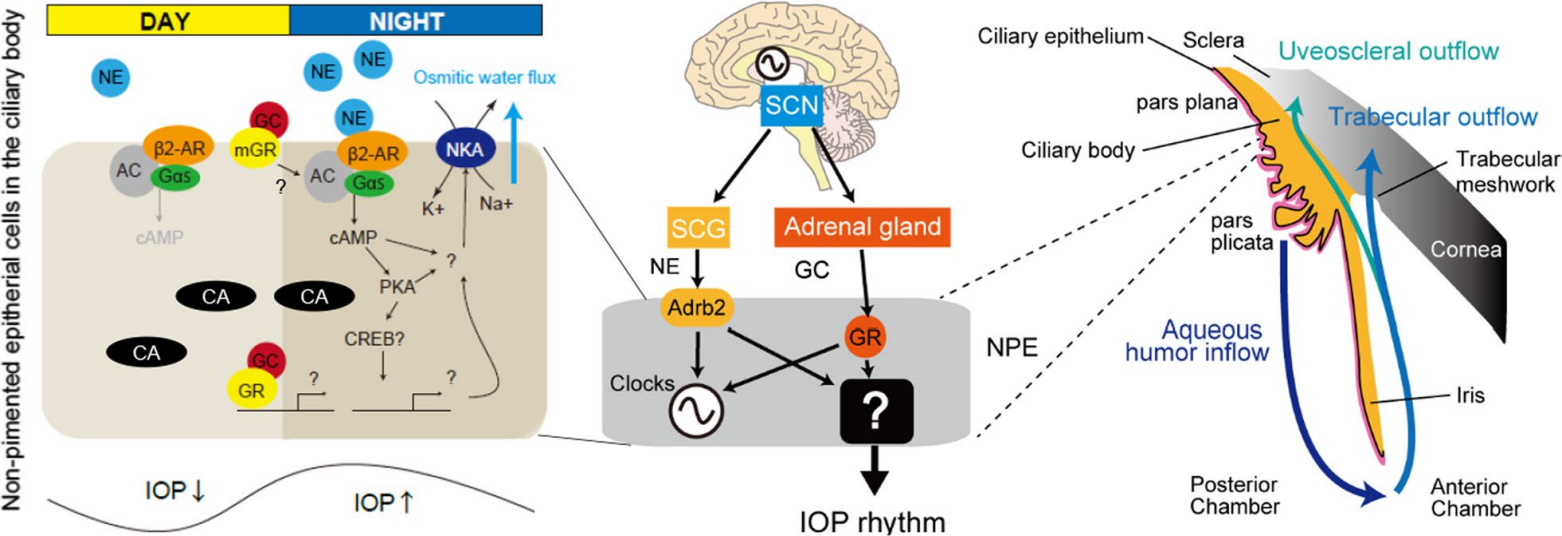
Regulatory pathways of IOP rhythm by the SCN and hypothesis model of aqueous humor inflow in mice. Regulatory pathways of the IOP rhythm by the SCN through the adrenal glucocorticoid and sympathetic nerve pathways and an anatomical model. The circadian timing signals of the NE and GC are mainly transmitted to the trabecular meshwork cells (TM) and ciliary epithelium (pars plana and pars plicata, respectively) for aqueous humor production, which seems to be independent of the ciliary clock. The aqueous humor is drained via two pathways: trabecular and uveoscleral outflows. However, the molecular mechanisms underlying the circadian regulation of aqueous humor production and drainage remain unclear. In nonpigmented epithelial (NPE) cells, Na^+^/K^+^ATPase (NKA) may mainly contribute to AH inflow rhythm formation via nocturnal β2-adrenergic receptor (AR; Abrb2)-mediated cAMP accumulation, which may generate nocturnal IOP increase. The role of GC, with an evening peak in rodents, in AH inflow rhythm formation remains unclear; this may be mediated by the cAMP-related membrane GC receptor (GR) or nuclear receptor GR. This figure is modified from a previous study [20]

The SCN synchronizes with most peripheral tissues and cells through various complex pathways, primarily involving the autonomic nervous system and endocrine signals [3] (Fig. 1). NE, released from the superior cervical ganglion (SCG), which is a part of the sympathetic nervous system, transmits circadian timing signals to the ciliary body of the eye to regulate pupil size, among other functions [21]. Furthermore, for most peripheral tissues, glucocorticoids (GCs) secreted from the adrenal glands, via the hypothalamus–pituitary–adrenal axis-mediated SCN, act as strong endocrine timing signals because GC receptors (GRs) are expressed in most peripheral cell types [22].

It has been reported that adrenalectomy (ADX) partially attenuates nocturnal IOP increase in rabbits [23] and mice [20]. Superior cervical ganglionectomy (SCGX) also has a minor effect on the IOP rhythm in rabbits [24] and mice [20]. However, these studies did not explain the signal pathways that generate the IOP rhythm. To address the relationship between glucocorticoids and sympathetic pathways in the IOP rhythm, we performed bilateral ADX and SCGX in mice [20]. ADX/SCGX suppressed the nocturnal IOP increase under both 12-h light 12-h dark conditions and constant dark conditions. The transmission pathway of the IOP rhythm seems to be composed of two components involving adrenal GCs and the sympathetic nervous system (Fig. 1).

### AH inflow rhythm

NKA and carbonic anhydrase CA [25] are ion transporters that play important roles in maintaining osmotic pressure balance during AH inflow. In our recent study, we found that the NKA antagonist ouabain, which is used in glaucoma therapy, when injected into the anterior chamber of the mouse eye, allowed for a nocturnal IOP increase, but prevented an overall IOP increase. This suggests a partial effect of NKA on AH inflow [26] (Fig. 1). Both glucocorticoid receptors (GR) and β2-adrenaline receptor (AR) are present in the pars plana of the ciliary NPE with weak expression in the retinal ganglion cell layer, inner nuclear layer, and pars plicata of the ciliary NPE in mice [20]. β1-AR is also localized in the NPE in mice [26]. Sympathetic nerve pathways regulate aqueous humor production [27]. The expression of β2-AR in the NPE of the human ciliary body was found to be higher than that of β1-AR [28]. NE, acting on β-AR, up-regulates the expression of the regulatory β1-subunit of NKA in neural cells [29]. Adrenaline, NE, and specific β2-AR agonists induce NKA activation in muscle via cyclic adenosine monophosphate (cAMP) [30] (Fig. 1).

In rat hippocampal slices, phosphorylation by the protein kinases PKG and PKC inhibits NKA activity, whereas dephosphorylation by the protein phosphatases PP-1 and PP-2B (calcineurin) reverses this effect in rats [31]. However, the NKA activation system in the ciliary body NPE remains unclear. In contrast, the effects of GC on the IOP inflow rhythm remain unclear. CAII co-localizes with GRs in the mouse brain [32], and dexamethasone (DEX) stimulation increases CAII expression. GR is also expressed in the human [33] and chicken ciliary bodies [34]. GC may regulate IOP rhythms by mediating AH production.

### AH outflow rhythm

**Fig. 2:**
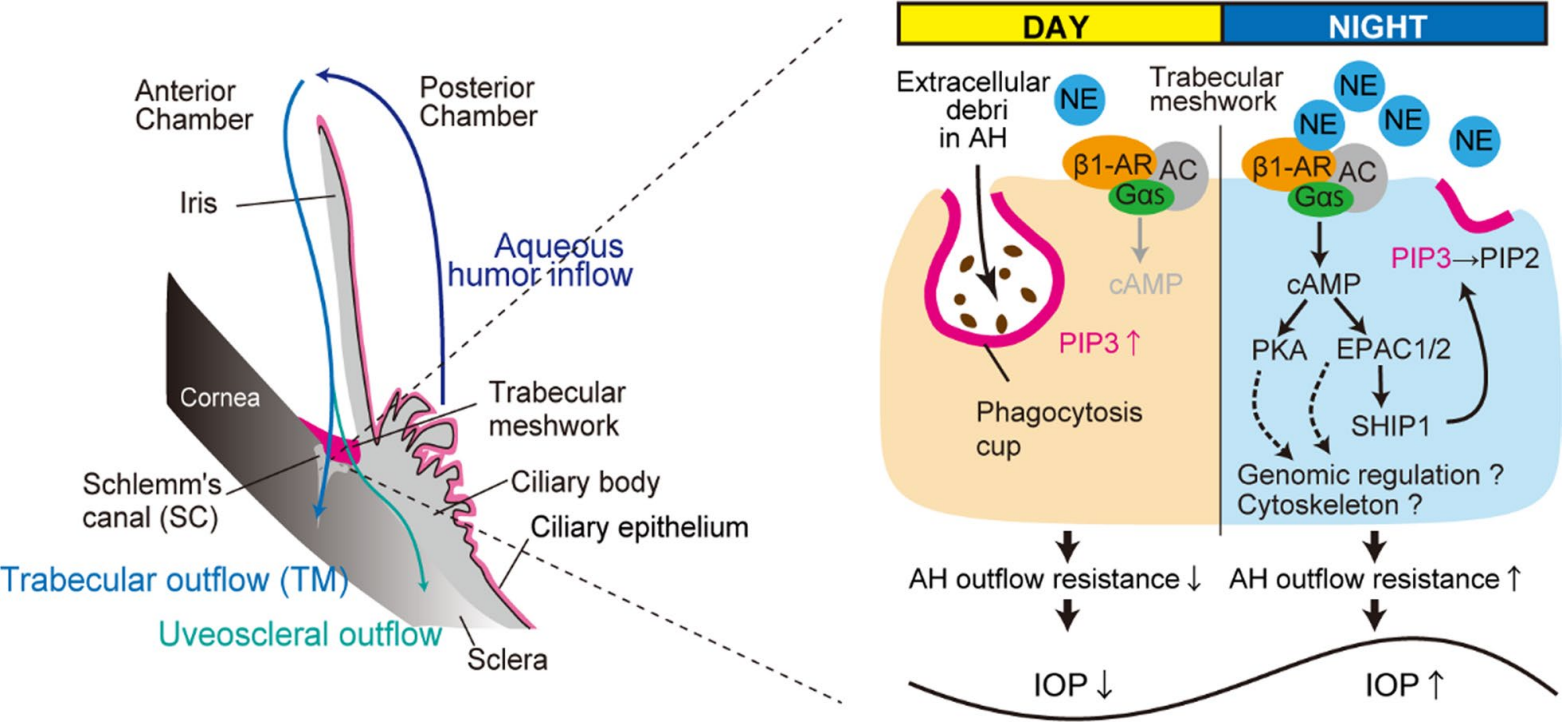
Time dependent regulatory model of AH outflow rhythm by sympathetic nerve NE. Regulatory model of time-dependent systems in which nocturnal NE suppresses phagocytosis of trabecular meshwork (TM) macrophage to induce AH outflow resistance through β1-AR-EPAC-SHIP1 activation. This nocturnal activation suppresses phagocytic cup formation and triggers PIP3 expression on the cellular membrane, leading to an increase in the IOP at night. Genomic regulation by NE and the effects of NE and GCs on the cytoskeleton in the TM remain unknown. This figure is modified from a previous study [26]

AH outflow studies have been conducted in rabbits [35] and mice [26]. AH outflow through the TM/SC may be involved in the daytime decrease of IOP (Fig. 2). In bead-injected eyes, the daytime IOP increased to the nocturnal level, and the day–night differences in individual IOP were halted by bead injection in rabbits [36, 37] and mice [26]. This suggests a daytime reduction in IOP due to AH outflow.

Phagocytosis and the remodeling of the actin cytoskeleton are closely related to AH outflow [6, 9–11]. Long-term treatment with DEX is known to reduce TM phagocytosis and increase stress on actin fibers in the human eye, as well as in primary TM cells [38, 39]. This leads to increased resistance in AH outflow and, ultimately, the development of glaucoma. Furthermore, NE suppresses the efficiency of macrophage phagocytosis in wound healing through α- and β-AR dependent pathways [40] and can promote actin polymerization in the retina [41]. Since all phagocytic processes rely on a finely controlled rearrangement of the actin cytoskeleton [42, 43], it has been challenging to isolate and study the effects of these two factors separately. In contrast, the regulation of AH outflow by the cytoskeleton appears to be limited to the nighttime or is time-independent, while TM phagocytosis seems to play a role in the daytime increase in AH outflow. Blocking of small GTPase RHOs or effectors of the RHO (ROCKs) decreases IOP not only by relaxing actin cytoskeleton but also by activating TM phagocytosis [9–11]. Studies in mice have shown that the reduction in IOP achieved with a ROCK inhibitor is limited to nighttime [26], and the blocking of RhoA by AAV (adeno-associated virus) intraocular injection prevents nocturnal IOP elevation in rats [44]. RHO may increase nocturnal TM phagocytosis and decrease nocturnal TM actin polymerization.

### Roles of NE and GCs in AH outflow rhythm

NE suppresses wound macrophage phagocytic efficiency [40]. We observed a significant suppression of phagocytosis in TM cells when exposed to NE. While GC alone had no effect, co-administration of GC with NE resulted in a dose-dependent suppression of phagocytosis, consistent with previous in situ studies [38, 39]. GC has been shown to bind to TM in humans [45], and its receptor localizes in mouse TM [20]. Thus, the interaction between GCs and NE can generate an appropriate AH drainage rhythm.

NE suppresses wound macrophage phagocytic efficiency through α- and β-AR dependent pathways [40]. In TM macrophages, β1-AR primarily mediates the effects of NE on phagocytosis [26]. β1-AR is a typical G protein-coupled receptor (GPCR) that preferentially binds to stimulatory G protein Gs to induce cAMP production. cAMP, in turn, activates protein kinase A (PKA) or exchange proteins directly activated by cAMP (EPACs) [46]. In microglia and peritoneal macrophages, myelin phagocytosis occurs with the involvement of both EPAC1 and PKA [47]. NE and the β-AR agonist isoproterenol suppress the phagocytosis of microglia cells via EPAC activation [48]. In our recent study, we found that Gs-coupled β1-AR modulates phagocytosis via PKA and EPAC in immortalized human TMC cells [26] (Fig. 2).

In general, β-AR blockers, including betaxolol, effectively reduce intraocular pressure (IOP) by decreasing aqueous humor (AH) inflow in patients [49]. However, several studies have demonstrated the opposite effect of the sympathetic role in AH outflow [50, 51]. β-ARs may be involved in increasing AH outflow by reducing the size of cells in the TM [50]. Continuous electrical stimulation of the cervical sympathetic nerve decreased IOP only for 1 h [51]. However, these studies specifically demonstrated the role of β2-AR. In fact, primary open-angle glaucoma (POAG) involves a greater increase in IOP at night when superior cervical ganglion-norepinephrine (SCG-NE) release is thought to be higher than during the day [16]. Furthermore, a recent study revealed that betaxolol is the top compound most opposed to POAG signatures, as calculated by microarray database analysis [52]. Β1-Ars may contribute to nocturnal AH outflow resistance.

β1-AR-mediated PI(3,4,5)P3 (PIP3) appears to modulate phagocytosis, which is necessary for this process [53]. The PIP3 antagonist, PITenin-7, dose-dependently and significantly suppresses the phagocytic activity of TM cells [26]. PI3K produces PIP3 from PI (4,5)P2 [54]. Class I PI3K has four isoforms: α, β, γ, and δ. PI3Kγ, but not α, β, and δ, modulates the phagocytosis of microglia, and this effect is suppressed by cAMP-mediated EPAC activation [55]. The selective PI3Kγ inhibitor, CAY10505, significantly restrains phagocytosis in TM cells [26]. Furthermore, the β1-AR agonist dobutamine decreases PIP3 levels in TM cells [26]. In macrophages, PKA does not inhibit phagocytosis, whereas Epac1 exerts an inhibitory effect mainly through the activation of the tyrosine phosphatase SHIP1 [56]. SHIP1 converts PIP3 to PI(3,5)P2 [54]. In a recent study, we found that dobutamine increases the phosphorylation of SHIP1, which was prevented by EPAC inhibition, but not by PKA inhibition, indicating the importance of β1-AR-mediated SHIP1 activation through EPAC [26]. We also found that Adrb1 and Ship1 are colocalized within the endothelial cells in the SC [26]. In addition, activated SHIP1 has been reported to dephosphorylate PTEN, catalyzing PIP3 in macrophages [56]. Single-cell RNA-seq analysis has suggested that both SHIP1 and PTEN are expressed in human TM macrophages [57].

Furthermore, PIP3 stimulates AKT and ERK1/2 signaling to modulate phagocytosis in circulating macrophages [53, 56]. In the TM of POAG donors, the cAMP signaling pathway and CREB were activated [58], while ERK phosphatase activity was downregulated [59]. In our recent study, we found that dobutamine cleanly inhibited AKT and ERK1/2 activations, which recovered significantly with EPAC inhibitor, PTEN inhibitor, and SHIP1 inhibitor, but not with PKA inhibitors. This indicates that, at least in vitro, β1-AR-mediated SHIP1 activation through EPAC appears to reduce PIP3 to suppress phagocytic cup formation (Fig. 2). The β1-AR antagonist betaxolol is well-used for glaucoma therapy [60], lowers mouse IOP topically [61], and blocks nocturnal IOP increase in mice [26]. In our recent study, we also found that instillation of EPAC and SHIP1 inhibitors to mice eye prevents nocturnal IOP increase, suggesting a role for the β1-AR-EPAC-SHIP1 pathway in vivo [26] (Fig. 2).

### Cytoskeleton, cell adhesion, and uveoscleral outflow

Long-term stimulation of GC increases the actin polymerization of TM and induces fibrosis [62]. In the TM, cAMP/PKA activation and downstream RhoA inactivation lead to a loss of actin stress fibers and focal adhesions, and disassembly of the matrix network [63]. *Ship1*−/− in neutrophils upregulates basal actin polymerization [64]. However, the time-dependent efficacy may explain their contribution to some extent. The effects of actin polymerization inhibitors on IOP reduction seem to be limited to the dark period in mice [26]. In contrast, in mice, 30–42% of AH passes through the uveoscleral pathway [65]. Latanoprost, a prostaglandin analog, decreases IOP by increasing the uveoscleral outflow of the aqueous humor in a time-dependent manner [66, 67]. To fully understand the AH dynamic rhythm, these determinants need to be elucidated.

## Importance of AR on IOP rhythm generation

It is certain that β1/2-ARs are not essential for IOP rhythm formation [13]. The removal of SCG in mice attenuated the IOP rhythm [20], and β1-AR antagonist betaxolol instillation resulted in IOP rhythms with a nocturnal peak flattening in both POAG and normal-tension glaucoma patients [60]. The contribution of the β1-AR-mediated circadian rhythm of AH resistance to IOP rhythm formation may not be as high, and DEX or the other AR can regulate AH outflow/inflow. β2-AR- or GC-mediated AH production in the NPE of the ciliary body is related to the IOP rhythm [20]. This may explain why β-AR1/2 double-knockout mice maintain this IOP rhythm, indicating GC-mediated IOP rhythm formation systems.

### Local clock in IOP rhythm

**Fig. 3:**
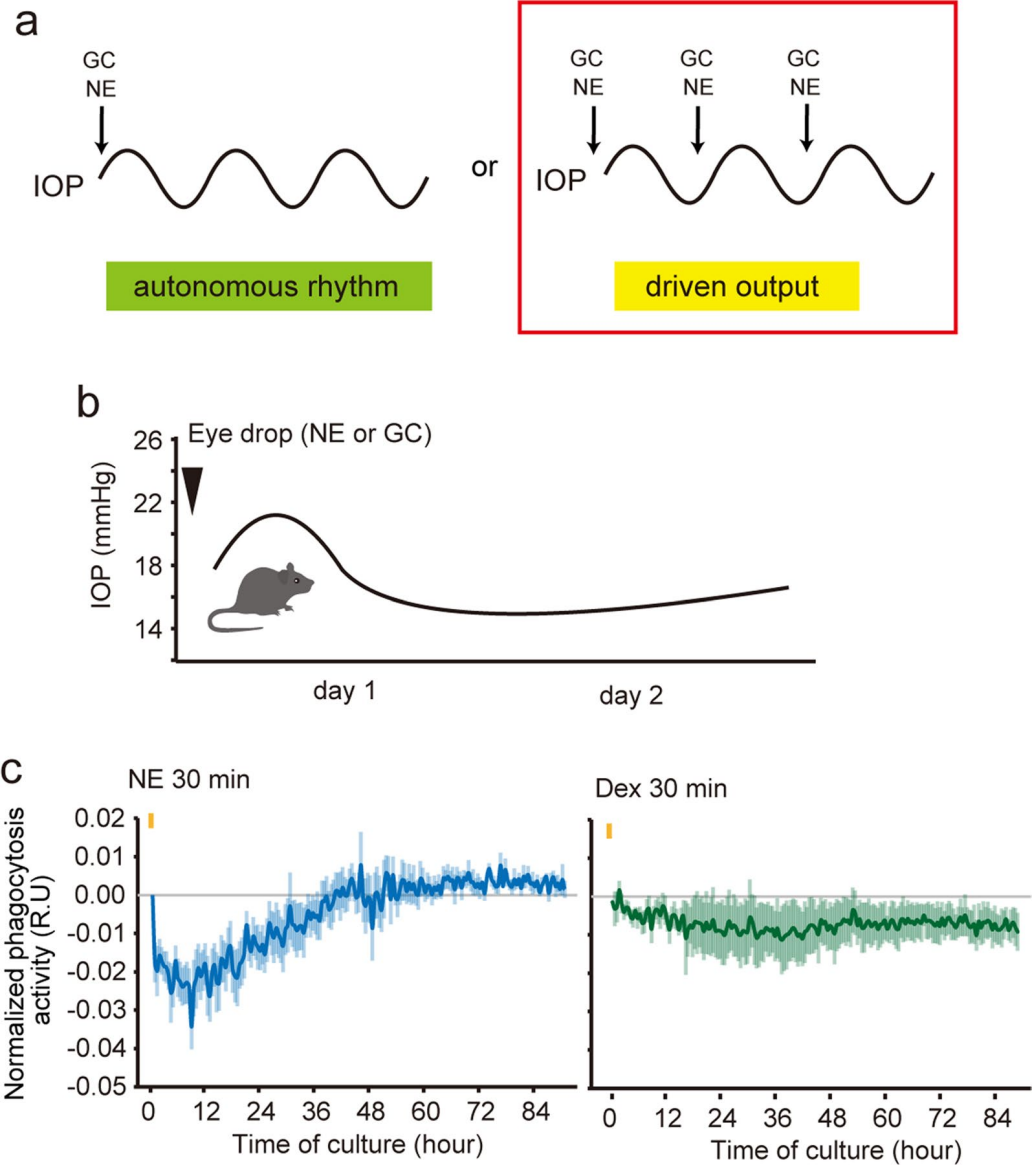
Driven-output stimuli generates circadian IOP rhythm. **a** Circadian time signals can generate rhythmicity through a self-sustainable autonomous rhythm by a single stimulation or a driven-output system by stimulation of circadian factors such as NE and GCs once a day. IOP rhythms may be independent of circadian clock regulation and may not be self-sustaining after a single stimulation. **b** Instillation of NE and GC induced a diurnal IOP rhythm on the 1st day, which disappeared on the 2nd day. **c** Exposure of TM cells to NE or dexamethasone (Dex) stimulation for 30 min and real-time phagocytic activity monitoring for 3 days. Circadian phagocytic rhythms were not detected. This figure was modified from previous studies [20, 26]

Circadian time signals can generate rhythmicity through self-sustaining autonomous rhythms with a single stimulation or a driven-output system by stimulating circadian factors such as NE and GCs once a day (Fig. 3A, B). In our recent study, changes in IOP induced by GC and NE were not sustained until the second day after a single instillation, indicating that IOP rhythms may be independent of circadian clock regulation and are not self-sustaining after a single stimulation (Fig. 3A, B). Moreover, the dispersed phase caused by ADX/SCGX was phase-advanced due to morning NE and GC instillation, when compared to the sham operated mice.

Real-time monitoring of the iris/ciliary body rhythm revealed disruption in the circadian rhythm of the iris/ciliary body caused by ADX and SCGX. Although the cultured iris/ciliary body rhythm cannot be entrained by light [14], it could be induced and entrained by DEX administration [68]. The rhythms, even in ADX and SCGX mice, were self-sustaining after a single DEX stimulation, in contrast to the IOP rhythm after DEX instillation [20], indicating independent regulation of the IOP rhythm by the local clock. ADX, along with NE-receptor blockers, induces a phase delay in the kidney and liver rhythm [69]. It is plausible that GC and NE act additively on the ciliary body to generate rhythmicity and may achieve an appropriate phase of IOP rhythm.

The IOP rhythm in *Cry1/Cry2*-knockout (KO) mice is markedly disrupted [70]. The circadian clock protein BMAL1, one of the most important clock proteins, is expressed in the NPE, mainly in the pars plana, but not in the TM. Retina–ciliary epithelium-specific Bmal1 KO mice (cKO) we generated with normal circadian rhythmicity of locomotor activity [71] maintained IOP rhythm [20]. The entrainment of the IOP rhythm may be directly regulated by GC or NE and not via the ciliary clock. This clock-independent regulatory pathway is consistent with the circadian regulation of the pineal gland [72]. The circadian rhythms of osteoclast-related genes were regulated by GC, independent of the local clock [73].

Similarly, we can observe the same trends in AH outflow. Acute NE stimulation suppresses TM phagocytosis only once, indicating the possibility of a driven-output phagocytic activity rhythm [26] (Fig. 3C). Furthermore, SHIP1 inhibition by a single instillation to the mice eye did not show any inhibitory effect on nocturnal IOP increase the day after instillation, providing evidence of driven-output IOP enhancement by nocturnal NE. This regulatory pathway is consistent with β1-AR-mediated circadian regulation of the pineal gland [72]. Since NE from the SCG appears to show a nocturnal peak in rodents [74], the circadian rhythm of SCG-NE can regulate diurnal changes in TM phagocytosis.

### Circadian rhythm of IOP in diurnal and nocturnal animals

The present model fails to fully account for the variations in IOP rhythms observed in diurnal and nocturnal animals. In nocturnal animals like mice, rabbits, and cats, the IOP rhythm peaks early at night [13, 14, 75], while in healthy diurnal humans, it tends to rise during the night and reaches its peak from late night to early morning [12, 15, 76]. The differences in the action of phasic SCG-NE and anti-phasic GC may contribute to the observed variations in IOP rhythms.

Although our model proposes an induction of IOP in response to nocturnal NE in nocturnal animals, the circadian rhythm of NE release from the SCG in humans remains unclear. Despite GC secretion peaking at light offset [77] and NE being released with a nocturnal peak from the SCG in rodents [74], GC rhythms are anti-phasic rather than SCG-NE in diurnal animals [74, 78]. Nocturnal NE release from SCG generally stimulates melatonin synthesis in humans and other mammals [79]. In humans, β-blockers inhibit nocturnal melatonin levels [80] and suppress IOP increase during late night to morning [81]. Furthermore, in nocturnal rabbits, β-blockers suppress IOP increase only at night [82]. These differences might be explained by oppositely phased rhythmic outputs from the SCN between species.

### Interaction with NE and GCs

The physiological implications of the dual regulatory pathways involving GC and NE remain unknown. However, considering that GC levels increase through diet [83], a sympathetic nervous pathway may serve as a backup. Both β-adrenergic signaling and GCs are known mediators of circadian rhythm output from the SCN to osteoblasts [84]. There could be interactions between GC and sympathetic pathways, as evidenced by our study where NE or GC instillation alone in mice eyes could not fully generate the amplitude of the IOP rhythm [20]. SCGX in rats suppresses the circadian GC rhythm in blood [85], and Adrb2 can regulate GR transactivation [86]. In contrast, GCT promotes the expression of several genes through glucocorticoid-response elements (GREs) [87], but it modulates *Adrb2* expression through the promotor GRE [88]. Moreover, ADX combined with NE-receptor blockers completely blocks exercise-induced entrainment of locomotor activity [69]. Therefore, the interaction between GC and NE could be crucial in generating an appropriate IOP rhythm.

Furthermore, NE is a necessary condition for GC-mediated acute phagocytosis suppression in TM cells. Interestingly, GCs rapidly activate cAMP production via Gαs to initiate non-genomic signaling, contributing to one-third of their canonical genomic effects [89]. Betaxolol, in fact, prevented steroid-induced IOP increase [90]. Thus, in TM, Gs-bound GR may enhance the β1-AR-Gs signal to suppress phagocytosis and generate an appropriate AH drainage rhythm.

## Conclusions

The transmission pathway of the IOP rhythm is composed of two circadian time signals derived from the circadian pacemaker SCN and involves adrenal GCs and the sympathetic nervous system. The AH inflow rhythm may be regulated by the ion transporter NKA in the NPE of the ciliary body via β2-AR and GR. The AH outflow rhythm can be produced by nocturnal NE-suppressed TM phagocytosis through the β1-AR-EPAC-SHIP1 pathway. Both AH dynamic systems seem to be independent of the local clock and may be driven output systems. In addition, the synergistic regulatory mechanism of GC in AH dynamics in the presence or absence of NE remains unknown. Since genomic signaling may also contribute to this mechanism [89], further understanding of the time-dependent efficacy of GC and NE on AH inflow/outflow will lead to the complete elucidation of the regulatory mechanisms of the IOP rhythm. Further understanding of this pathway is necessary to fully explain the complex cellular mechanisms by which the occupancy of specific ARs and GR regulates AH dynamics, which may contribute to the establishment of chronotherapy and the development of multiple types of drugs.

## Abbreviations

AH: Aqueous humor
SCN: Suprachiasmatic nucleus
NE: Noradrenaline, norepinephrine
GC: Glucocorticoids
IOP: Intraocular pressure
AR: Adrenergic receptor
GR: Glucocorticoid receptor
cAMP: Cyclic adenosine monophosphate
ROCK: Rho-associated coiled-coil-containing protein kinase
SCG: Superior cervical ganglion
SCGX: Superior cervical ganglionectomy
ADX: Adrenalectomy
DEX: Dexamethasone
POAG: Primary open-angle glaucoma
NKA: Na^+^/K^+^-ATPase
CA: Carbonic anhydrase
